# Characterization of Retinal Functionality at Different Eccentricities in a Diurnal Rodent

**DOI:** 10.3389/fncel.2018.00444

**Published:** 2018-12-03

**Authors:** María-José Escobar, César Reyes, Rubén Herzog, Joaquin Araya, Mónica Otero, Cristóbal Ibaceta, Adrián G. Palacios

**Affiliations:** ^1^Departamento de Electrónica, Universidad Técnica Federico Santa María, Valparaíso, Chile; ^2^Centro Interdisciplinario de Neurociencia de Valparaíso, Universidad de Valparaíso, Valparaíso, Chile; ^3^Programa de Doctorado en Neurociencia Universidad de Santiago de Chile, Santiago, Chile

**Keywords:** retina, MEA, central vs. periphery, RGCs, spatiotemporal analysis, receptive field properties

## Abstract

Although the properties of the neurons of the visual system that process central and peripheral regions of the visual field have been widely researched in the visual cortex and the LGN, they have scarcely been documented for the retina. The retina is the first step in integrating optical signals, and despite considerable efforts to functionally characterize the different types of retinal ganglion cells (RGCs), a clear account of the particular functionality of cells with central vs. peripheral fields is still wanting. Here, we use electrophysiological recordings, gathered from retinas of the diurnal rodent *Octodon degus*, to show that RGCs with peripheral receptive fields (RF) are larger, faster, and have shorter transient responses. This translates into higher sensitivity at high temporal frequencies and a full frequency bandwidth when compared to RGCs with more central RF. We also observed that imbalances between ON and OFF cell populations are preserved with eccentricity. Finally, the high diversity of functional types of RGCs highlights the complexity of the computational strategies implemented in the early stages of visual processing, which could inspire the development of bio-inspired artificial systems.

## 1. Introduction

The visual system integrates optical signals to create specific features of the images such as movement, contrast, and color (Van Essen et al., 1992; Masland, 2012). Although some studies suggest that there exists within the visual cortex a functional and modular segregation of regions that attend to the central vs. peripheral visual field (Stone, 1983; Orban et al., 1986; Loschky et al., 2017), the existence of such segregation at the level of the retina remains hardly studied. On the other hand, studies have described that any point of a visual scene is anatomically covered through a variety of different RGC types (for example, parasol and midget RGCs in primates or alpha cell RGCs in rodents) that form potential visual channels (e.g., ON / OFF) acting as functional filters for the physical world (Field and Chichilnisky, 2007). Moreover, Polyak (1957) raised what is still an unresolved problem how the diversity of RGCs contributes to forming a variety of visual channels as a function of retinal eccentricity, which is vital for many species (Boycott and Wässle, 1974; Gollisch and Meister, 2010).

Most of the functional segregation of RGCs comes from the structural organization of the retina, which is stratified into nuclear and plexiform layers, involving several classes of visual cells (photoreceptors, bipolar, horizontal and amacrine cell, ganglion cells) that differ in their morphology and physiology. For example, OFF-bipolar cells (BCs) hyperpolarized as light intensity increased through AMPA or Kainate receptors (Devries, 2000). By contrast, ON-BCs depolarized as light intensity decreased, through MGluR6 receptors. Axons of BCs project into the inner plexiform sublayer and pass their ON / OFF signature to different types of RGCs.

In mammals, RGC types have been reported that differ in morphology, gene expression, and physiological properties (Rockhill et al., 2002; Sanes and Masland, 2015). The classical categories for cats proposed by Enroth-Cugell and Robson (1966), distinguish X (sustained) and Y (transient) RGCs, while in primates they correspond to midget and parasol monostratified cells (Croner and Kaplan, 1995), which represent 80 and 10% of the entire population of RGCs and form the magnocellular and parvocellular visual streams, respectively (Dacey, 2000). In mice, the α-RGCs correspond to RGCs with large dendrite fields (Peichl et al., 1987; Peichl, 1991). Moreover, four types of α-RGCs in mice (Pang et al., 2003; Krieger et al., 2017) have been reported to have different computational properties.

Rodents are a compelling model for the study of the anatomy and physiology of RGCs (Sun et al., 2002; Wässle, 2004; Masland and Martin, 2007; Sanes and Masland, 2015). For example, the guinea pig retina shows different types of RGCs: brisk-transient; brisk-sustained; local edge; direction and motion selective (Demb et al., 2001), and a brisk-transient ON/OFF coding for contrast sensitivity (Zaghloul et al., 2003). Interestingly, their retina presents a higher number of OFF-RGCs compared to ON-RGCs (Ratliff et al., 2010), which forms functional mosaics that favor a precise analysis for contrast under natural viewing conditions (Devries and Baylor, 1997), similar to what has been reported for primates (Gauthier et al., 2009) and rodents (Anishchenko et al., 2010; Yu et al., 2017).

Although different RGC types seem to pave the entire retinal mosaic, their functional characterization can vary according to several factors including retinal eccentricity, development, lifestyle, or specific behaviors (Anishchenko et al., 2010; Masland, 2017; Sinha et al., 2017). Recently, a sophisticated clustering and genetic analysis in mice has extended the diversity of RGCs to a much higher number than expected before, posing a question on the generality of such results for other species (Sümbül et al., 2014; Baden et al., 2016).

Studies on the visual system of diurnal mammals are scarce. For example the *Octodon degu* rodent (Chávez et al., 2003) shows a dichromatic color vision system with isodensity zones where the highest concentration of RGCs is in the central retina (Vega-Zuniga et al., 2013), apparently aligned with the spatial distribution of retina photoreceptors (Jacobs et al., 2003). This distribution of photoreceptors and RGCs along visual regions motivated the present study. Here we characterized RGCs from central and peripheral retina regions and, in particular, examine whether any functional asymmetries are present between ON and OFF cell populations along the eccentricity axis.

## 2. Materials and Methods

### 2.1. Multielectrode Recording and Animal Preparation

**Figure 1:**
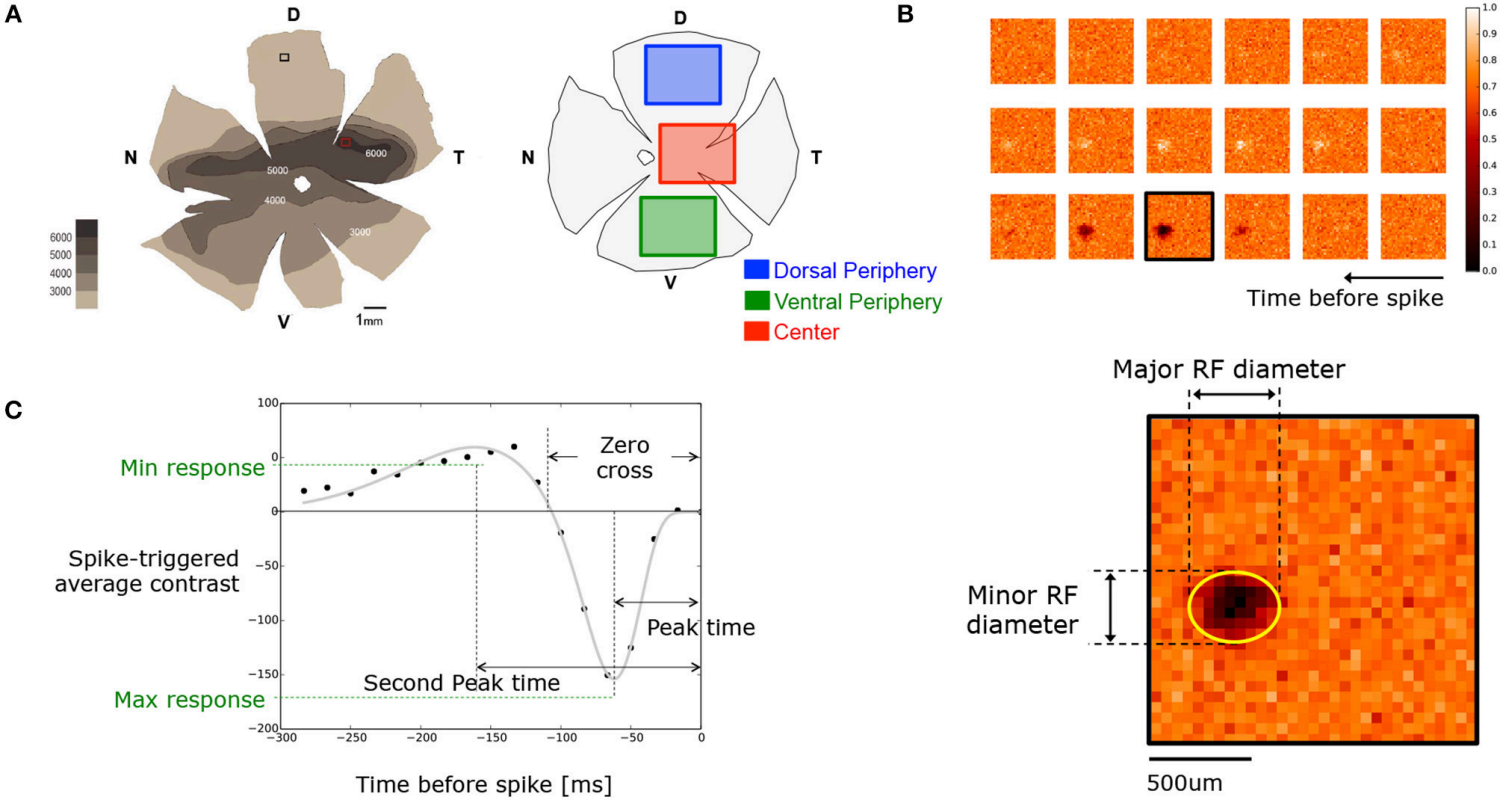
**(A)** (Left) *Octodon degu's* retinal wholemount showing the RGC layer density map for the left eye retina reported by Vega-Zuniga et al. (2013). (Right) Retina diagram specifying the dissected regions considered as central and peripheral retina. Notice that peripheries could come from dorsal or ventral regions. **(B)** Sample of the time course STA obtained for an OFF RGC. Parameters used for the spatial characterization, such as minor and major RF diameters, were obtained by fitting an ellipse on the frame with the highest activity. **(C)** Temporal time course of the same OFF cell shown in **(B)**. Each black dot represents the intensity around the center of the RF for each frame. Gray line shows the resulting fitting curve (see Equation 1). From the fitted version we extracted parameters such as peak-time, second peak-time, zero-cross, and maximal and minimal response.

The animal care and experimental methodology used here has already been described in Palacios-Muñoz et al. (2014). In brief, young adult (3–8 months old) *Octodon degus* (degus) born in captivity were maintained in large collective cages in an animal facility at 20–25°C under a 12 / 12-h light-dark cycle, with access to pellet food and water *ad-libitum*. A bioethics committee, under the regulation of the Chilean Research Council (CONICYT) at the Universidad de Valparaiso, approved the experimental protocol. Before each experiment degus were dark adapted for 30 min and then deeply anesthetized with Isoflurane (Sigma-Aldrich Co.), then euthanized by decapitation once vital reflexes were absent. Eyes were quickly enucleated and eye-cups prepared under dim red light and immersed in bicarbonate-buffered Ames Medium (Sigma-Aldrich Co.) and kept continually oxygenated (95% + 5%) at room temperature. The retina pieces studied were dissected from central and periphery zones (Figure 1A) and carefully separated from the retinal pigment epithelium. The dissected pieces were then mounted on a ring containing a dialysis membrane (Spectra MWCO 25,000) and fit in an up/down plastic cylinder that was adjusted to contact the RGC layer with the surface of the multi-electrode recording array (MEA) system (Multichannel Systems, 16x16 electrode grid model 256MEA100/30iR-ITO, electrode diameter of 30 and 100 [μm] between electrodes) for *in vitro* spike recording from RGCs.

### 2.2. Visual Stimulation

Visual stimuli were generated using customized software (using PsychoToolbox MatLab) and projected onto the retina using a LED projector (PLED-W500, ViewSonic, USA) equipped with an electronic shutter (Vincent Associates, Rochester, USA). The optical system included a series of optical density filters to control light intensity. The MEA preparation was placed on an inverted microscope (Lens 4, Eclipse TE2000, Nikkon, Japan) fitted with a CCD camera (Pixelfly, PCO, USA) for visualization and stimulus calibration.

We projected images of 372 × 372 pixels, where each pixel covered a surface of 16 μm^2^. Degus are crepuscular dichromatic (green and blue/UV cones) animals Chávez et al. (2003), thus, only B (blue) and G (green) beams of the projector were used for visual stimulation, while the R (red) channel was used for signal synchronization. Checkerboards were formed by black combined with B and G colors with a bin size of 50 μm at a rate of 60 fps for 30 min. In addition, we stimulated with full-screen light flashes for a period of 1,400 ms divided in 400 ms ON and 1,000 ms OFF. The light flash sequence was repeated 30 times.

### 2.3. Spike Sorting and Data Analysis

Extracellular potentials were recorded by using the MC/Rack software (MultichannelSystem, Germany) and data analyzed with the SpykingCircus software (Yger et al., 2016). This software can sort the spikes from hundreds of neurons simultaneously, which are checked using a dedicated GUI to remove duplicates. The spikes obtained were validated using interspike interval (ISI) and through autocorrelation analysis criteria. They were also checked through cross-correlation with previously obtained templates. Templates with a high cross-correlation and similar receptive field (RF) characteristics and light responses were merged.

### 2.4. Receptive Field Estimation

We collected data from five retinal pieces (5 different animals), two central and three peripheral regions, which included a total of 761 RGCs: 341 for center and 420 for the periphery. For estimating the RF, a checkerboard white noise stimulus was used. The spatiotemporal RF of each cell was determined using custom-built software to perform a Spike Triggered Average (STA) (Chichilnisky and Kalmar, 2002) RF for each of the 761 RGCs recorded. The spatial component of the RF was obtained fitting a 2D Gaussian on the frame with the maximal response. The temporal component of the RF was recovered by collecting the average response of a window of 3 × 3 pixels around the center of the spatial RF along 18 frames before the action potential generation. The temporal profile was then fitted to the difference of two cascade low-pass filters, as shown in Chichilnisky and Kalmar (2002).

(1) $$\begin{array}{ll} f\left(t\right) & =a_{1}{\left(t/\tau_{1}\right)}^{n}\exp\left(-n\left(t/\tau_{1}-1\right)\right)\\ - & a_{2}{\left(t/\tau_{2}\right)}^{n}\exp\left(-n\left(t/\tau_{2}-1\right)\right). \end{array}$$

As the Fourier transform (FT) of (1) is defined, the fitted parameters were also used to obtain a temporal frequency domain representation for each RGC. The temporal FT of (1) is defined in terms of the temporal fitting parameters *a*_1_, *a*_2_, τ_1_, τ_2_, and *n*. The temporal frequency selectivity is obtained from the magnitude of the FT of (1), as the temporal frequency with the highest response. Similarly, the temporal frequency bandwidth was also obtained from the magnitude of (1), as the frequency bandwidth obtained at half of the maximal frequency response.

### 2.5. Light Response Characterization

RGCs were analyzed according to their response to flashing light stimulation. The RGC spike response was used to classify the neurons in ON, ON-OFF, or OFF cells. To do so, we calculated the Peristimulus Time Histogram (PSTH) with a bin size of 5 ms, and then computed the mean (μ) and the standard deviation (σ) response along the stimulus period. As the ON and OFF flashes have different time durations, we did all the computations considering the shortest period. Only units with a significant activity over δ = μ±1.5σ along the ON and OFF flashes were selected, and the threshold δ was also used to discard those cells with deficient activity (less than 20 spikes in all bins).

The response latency of each cell was computed as the time when the maximal firing rate is reached after the stimulus onset. The sustained index used to analyze different light-flash responses was computed as

(2) $$\begin{array}{l} SI=\frac{R_{max}-R_{mean}}{R_{max}+R_{mean}}, \end{array}$$

where *R*_*max*_ (*R*_*mean*_) is the maximal (mean) firing rates of the cell observed in a given condition.

To sort the different cell types according to their temporal response, we used the fitted curve, defined in (1), to extend the temporal characterization of the cell up to 40 frames prior to the spike generation (666 ms) with a resolution of 900 points. Starting from this curve, we calculated some temporal characteristics of the RF such as the peak time, representing the moment at the maximal contrast intensity; the zero-crossing of the curve, which stands for the difference between the generation of the spike and the zero-crossing time of the curve; and the peak amplitude, which corresponds to the STA contrast value at the peak time. Finally, the biphasic index is the absolute value of the ratio between the minimal and maximal amplitude of the STA time curve (see Figure 1C).

Temporal cell profiles were clustered using their five first principal components using principal component analysis (PCA). Following the approach proposed by Rodriguez and Laio (2014), which sets as centroids of the clusters the templates with the highest local density, we clusterized functional types for peripheral fragments of the retina. Once each group was obtained, we computed the mean of the temporal profiles for each of them as well as obtaining the parameters for the fitted curve defined in (1).

### 2.6. Linear and Non-linear Response

For each cell, the linear response was computed convolving the estimated RF with the checkerboard stimulus. The curve *n*_*l*_, relating the measured with the linear estimated response, was fitted by the following curve

(3) $$\begin{array}{l} n_{l}\left(x\right)=\alpha \log\left(1+\exp\left(1+\beta \left(x-\theta \right)\right)\right), \end{array}$$

where mainly the parameters β and θ quantify the non-linearity represented in the sigmoid function described in (3).

Additionally, similar to Chichilnisky and Kalmar (2002), we implemented the Non-Linear Index (NLI) defined as follows

(4) $$\begin{array}{l} \text{NLI}=\log\left(\frac{s_{\text{max}}}{s_{\text{zc}}}\right), \end{array}$$

where *s*_max_ refers to the slope of the non-linear response fitted by (3) at the value with the highest response, and *s*_zc_ the slope at zero crossing.

### 2.7. Mutual Information

Mutual information was used to compare the information captured by the simulated central and peripheral retinas. Let *L*(*x, y, t*) be the input sequence video which is convolved with a spatio-temporal filter, obtaining $\tilde{L}\left(x,y,t\right)$ video output. For a given position (*x, y*), we computed the probability distribution of the pixel intensity at that position along time, obtaining *p*_*L*_ and $p_{\tilde{L}}$ for *L*(*x, y, t*) and $\tilde{L}\left(x,y,t\right)$, respectively. The mutual information $I\left(L;\tilde{L}\right)\left(x,y\right)$ is then defined as

(5) $$\begin{array}{l} I\left(L;\tilde{L}\right)\left(x,y\right)=\underset{i\in \Phi_{L}}{\sum }\underset{j\in \Phi_{\tilde{L}}}{\sum }p_{L,\tilde{L}}\left(i,j\right)\log\left(\frac{p_{L,\tilde{L}}\left(i,j\right)}{p_{L}\left(i\right)p_{\tilde{L}}\left(j\right)}\right), \end{array}$$

where $p_{L,\tilde{L}}\left(i,j\right)$ denotes the joint probability of the two vectors, with domains Φ_*L*_ and $\Phi_{\tilde{L}}$ for *i* and *j*, respectively.

## 3. Results

This study was done in two steps. First, we showed the general behavior of the entire population of recorded RGCs, which may contain a variety of different functional types. Second, we isolated the fast-biphasic OFF RGC subtype in all the preparations, comparing now the properties of a single functional type present at different eccentricities. To characterize different cell populations of RGCs present in the degus retina, we collected a series of data coming from *in-vitro* extracellular MEA recordings stimulated with white-noise checkerboards. We analyzed five retinal preparations, dissected from regions following RGCs, isodensity distribution, as it was reported by Vega-Zuniga et al. (2013) (see Figure 1A left).

Using a white noise checkerboard as an input stimulus, we computed the spike-triggered average (STA) for each RGC, obtaining the temporal profile and spatial extent of their responses. RGCs were categorized as ON or OFF according to the polarity of their response (see Table 1). According to these values, we observed a tendency for the centers to contain fewer ON cells than peripheries, which have proportion values similar to the ones reported in (Palacios-Muñoz et al., 2014).

**Table 1:** Number and percentages of ON, OFF, and ON-OFF cells encountered in the retina preparations considered in this study.

	**Number of cells**		**Light flashes**			**Checkerboard**	
			**ON**	**OFF**	**ON-OFF**	**ON**	**OFF**
Center	Total	749	11.7 ± 10.4	23.4 ± 11.1	47.5 ± 4.0		
	Valid RF	341	6.3 ± 4.7	36.0 ± 2.7	48.3 ± 32	10.4 ± 1.0	89.6 ± 1.0
Periphery	Total	1690	9.7 ± 1.0	23.0 ± 1.6	49.7 ± 10.5		
	Valid RF	420	9.9 ± 2.4	40.9 ± 9.0	39.8 ± 9.5	15.84 ± 1.3	84.2 ± 1.3

In the case of light flashes, we indicate the proportion of the total number of cells, vs. the cells with a valid RF

### 3.1. Spatial Functional Properties

The RGC response to the white-noise checkerboard stimulus was used to compute the STA for each cell. As mentioned previously, we only considered cells with a valid STA in the analysis, i.e., cells where the sum of the first and second peak responses was over four times the estimated standard deviation. As it is shown in Figure 1B, the spatial extent of the RF was computed by fitting a 2D Gaussian to the temporal frame giving the maximal response.

**Figure 2:**
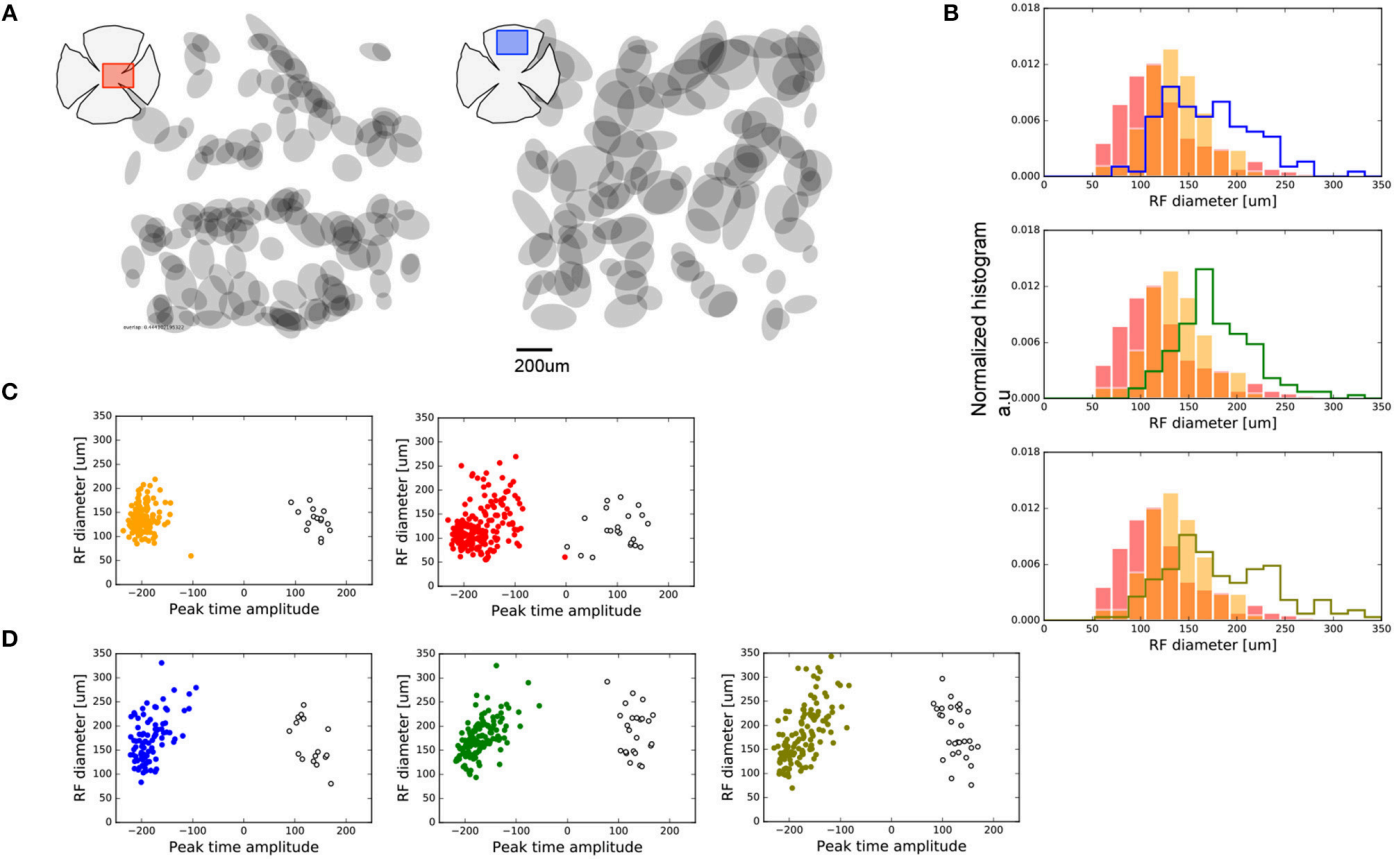
Spatial features observed for central and peripheral RGCs. **(A)** Sample of RGCs RF maps encountered in one sample of central (left-red) and peripheral (right-blue) retina studied. **(B)** Distribution of RF areas of the two central (red and orange bars) vs. all the peripheral retinas represented as stepped histograms. **(C)** Distribution of RF diameters of the central retinas regions separated in ON and OFF cell populations. ON cells, with a positive peak-time amplitude, are represented as empty black circles. Filled colored circles represent OFF cells. **(D)** Same as **(C)** for the peripheral retinas.

We analyzed the spatial properties of the central and peripheral dissected retinal regions, as shown in Figure 2. For each retina preparation, we computed the map of all the RGCs RF encountered (sample maps are shown in Figure 2A). We computed the RF diameter as the square root of the product between the major and minor RF radius. Each histogram in Figure 2B shows the distribution of the RF diameter found for each sample of retina. Red and orange colors represent data for the two central regions analyzed, and each histogram compares them against those for each of the three peripheral regions studied.

The population of RGCs recorded in our experiments exhibits larger RF for peripheral areas compared to the RFs for central regions of the retina. The RF diameters of RGCs found at the center differ between the two preparations (Kolmogorov-Smirnoff—KS, *p* < 1e-8); this was not observed for the preparations from peripheral regions (KS, *p* > 0.01). Nevertheless, both central regions contained cells with a RF diameter smaller than those from the periphery (KS, *p* < 1e-10).

In addition, we aimed to observe functional asymmetries between ON and OFF cell populations recorded in each retina preparation. Figures 2C,D show the distribution of RF diameters segregated by ON and OFF cell population obtained from the value of the peak-time amplitude parameter, for center and periphery, respectively.

### 3.2. Temporal Functional Properties

Temporal analysis obtained from STA reflected different cell dynamics at the central and peripheral retina. Each RGC's temporal course profile was obtained as the intensity value of the RF center along a time window of 400 ms prior to a spike. The temporal pattern was fit with the expression provided in the Methods (Chichilnisky and Kalmar, 2002), obtaining a parametric expression from which the values of interest can be extracted analytically.

**Figure 3:**
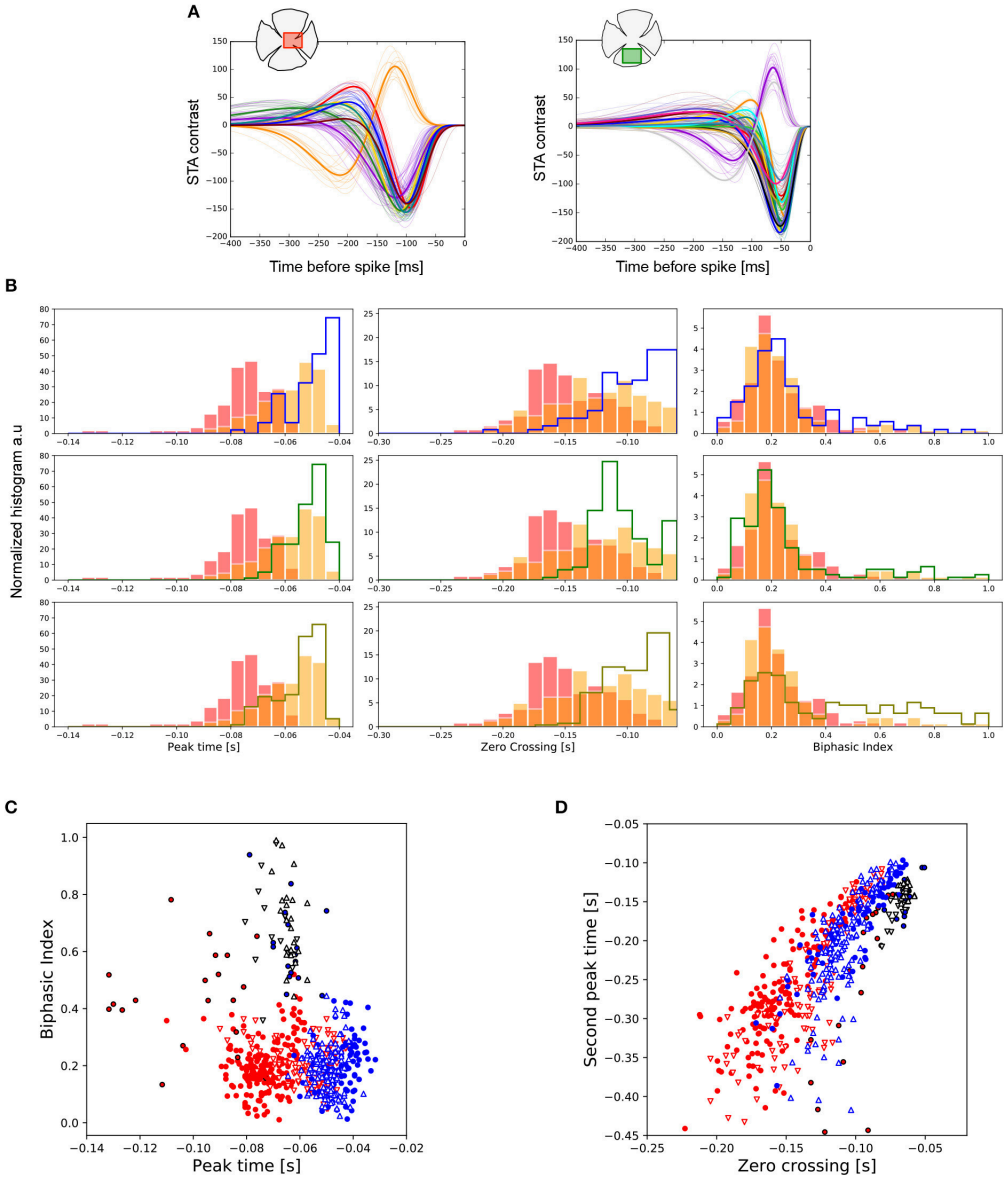
Temporal features observed in central and peripheral RGCs. **(A)** Temporal profile of all the RGCs from one central (left) and one peripheral (right) retina region. Each color line represents the associated cluster used to visually organize different functional cell types, whose mean curve is plotted with a thick colored line. The number of clusters encountered, from left to right, was 14 and 17, respectively. **(B)** Distributions of the peak-time (left), zero-cross (middle) and biphasic index (right) parameters for the total number of retina pieces analyzed. The data coming from the two centers are represented in red and orange, respectively. Each row represents a comparison against a different preparation from peripheral retina. **(C)** Chart represented the covariation of the peak-time response vs. the biphasic index for all the experiments analyzed. Central preparations are represented in red (◦ and △) and peripheries in blue (◦ and ∇). In all the cases, ON cells are indicated in black. **(D)** Same as **(C)**, but now showing the covariation of zero-crossing and second peak-time.

For both the central and peripheral regions of the retina we clustered the temporal profiles by experiment, obtaining the representation shown in Figure 3A. The color of the temporal profile indicates the associated cluster, and a thick colored line represents the average value. We show an example of only one center with 7 clusters (left), and a ventral periphery with 15 clusters (right). From the parametric expression obtained for each RGC's time course we extracted values of interest such as peak time, zero crossing and biphasic index (see Figure 1).

Figure 3B shows the comparison of the temporal properties of central vs. peripheral RGCs. The data obtained from central retinal regions are represented with filled bars in red and orange. Each row compares these data against those from a different preparation of the peripheral retina. The five preparations were statistically different concerning peak-time (left). Nevertheless, responses recorded for all the peripheral preparations were faster than for any of the central regions studied (KS, *p* < 0.01 in the worst case). The duration of the temporal response represented by the zero-crossing parameter (middle) was also shorter for RGCs located at the periphery (KL *p* < 1e-07 in the worst case). In the case of the biphasic index (right), only the periphery preparation on the last row showed statistical differences with both centers (KS, *p* < 0.001).

In addition to differences in the temporal properties of central vs. peripheral RGCs, differences were also observed between ON and OFF cell populations. Figure 3C shows a comparison between the temporal properties of the merged centers (red) and peripheries (blue) considering ON and OFF cell populations. OFF cells are represented in colored contours, while ON cells are represented in black contours. In addition, in all the retina preparations we observed an OFF cell population (colored contours) with lower peak-time values compared to ON cells (black contours) (KS, *p* < 1e-5 in the worst case). We found similar behavior with the biphasic index parameter: ON cells had larger biphasic index values compared to OFF cells, both for central and peripheral retina pieces (KS, *p* < 1e-6 in the worst case). Central retina preparations have higher asymmetries of the biphasic index between ON and OFF cell populations compared with peripheries.

ON cells have shorter zero crossing values compared to the whole group of OFF cells recorded (see Figure 3D) (KS, *p* < 1e-9 in the worst case). The second peak time parameter observed in the ON population presents shorter values compared to those of OFF cells (KS, *p* < 1e-5 in the worst case).

**Figure 4:**
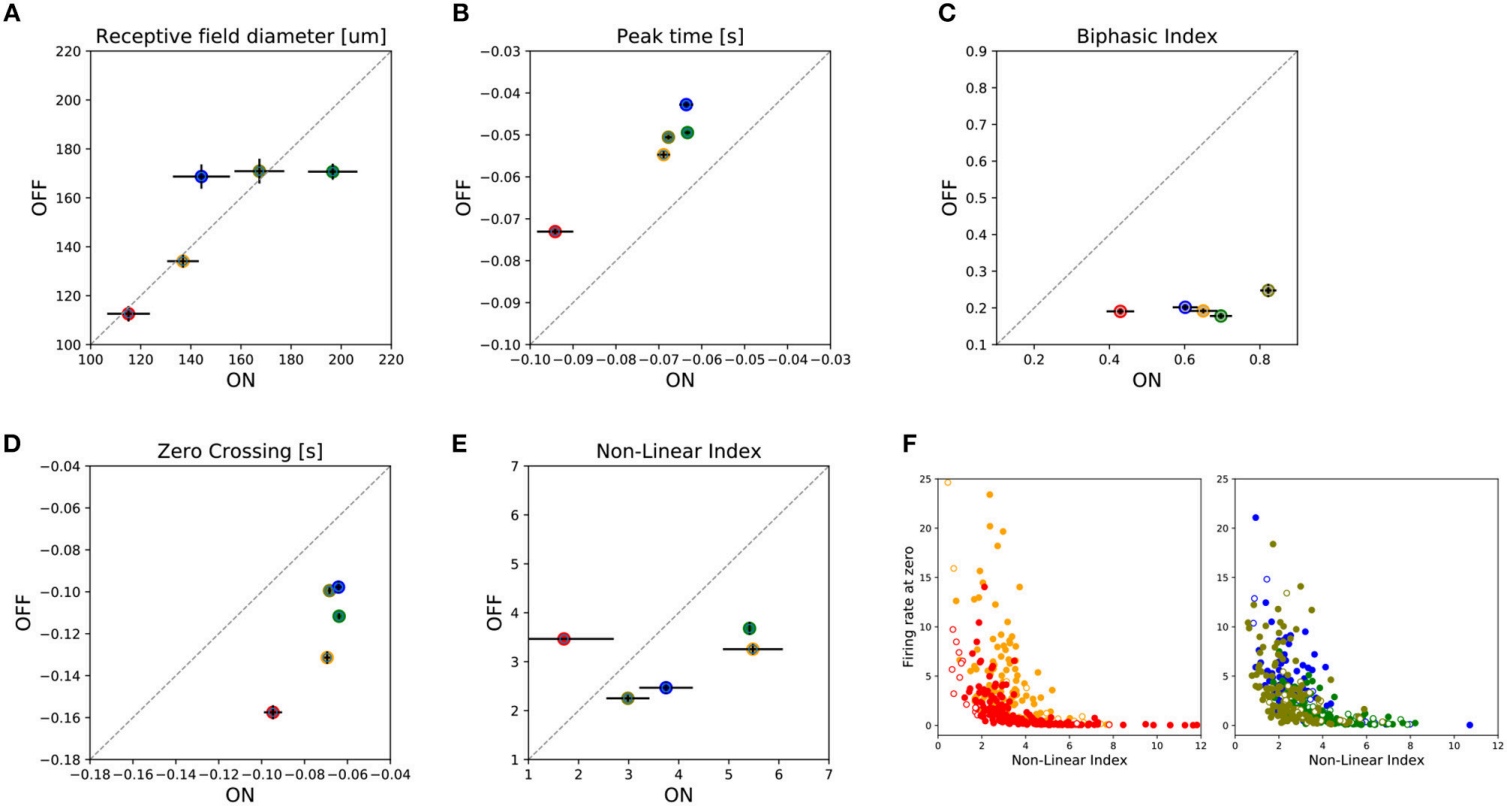
Summary of asymmetries observed between ON and OFF cells. For five retina preparations, two from central and three from peripheral regions of the retina, a total of 102 ON cells and 659 OFF cells were analyzed by grouping the information as shown in the figure. Filled circles represent the median value for that parameter for a given preparation, while horizontal/vertical bars represent the SEM value for the ON and OFF population, respectively. In the figure we have represented: **(A)** receptive field diameter; **(B)** peak-time; **(C)** biphasic-index, **(D)** zero-crossing parameters, and **(E)** the Non-Lineal Index. Finally, **(F)** shows the covariation between the non-linear index parameter and the firing rate measured in the cell when the estimated linear response is zero. Empty (filled) colored circles represent ON (OFF) cells. The left (right) panel shows the data for the central (peripheral) preparations.

A global comparison of the functional parameters of all the ON and OFF RGCs recorded per retina preparation is shown in Figure 4. From this figure, we observe that ON-OFF asymmetries, such as the biphasic index and peak time response, are preserved in all the preparations at different eccentricities. However, for the RF's diameter and zero crossing parameters we found asymmetries even for the same retinal region. This could be due to the obscuring effect of a global analysis that includes different RGC subtypes recorded in each retina preparation. Nevertheless, we do not expect to find differences in the RF diameter of ON and OFF RGCs belonging to the same type. Figure 4E represents the Non-Linear Index (NLI) computed for all the retina preparations (see section 2), indicating the ON and OFF asymmetries. Figure 4F presents the covariation of the non-linear index, and the firing rate obtained when the linear estimated response obtained by STA is zero. The left panel represents central retina preparations while the right panel shows the peripheries.

### 3.3. Frequency Selectivity of the Central and Peripheral Retina

This frequency analysis aims to correlate time domain feature parameters with temporal frequency characterization, and thus to have an interpretation of RGC receptive fields as image processing filters.

**Figure 5:**
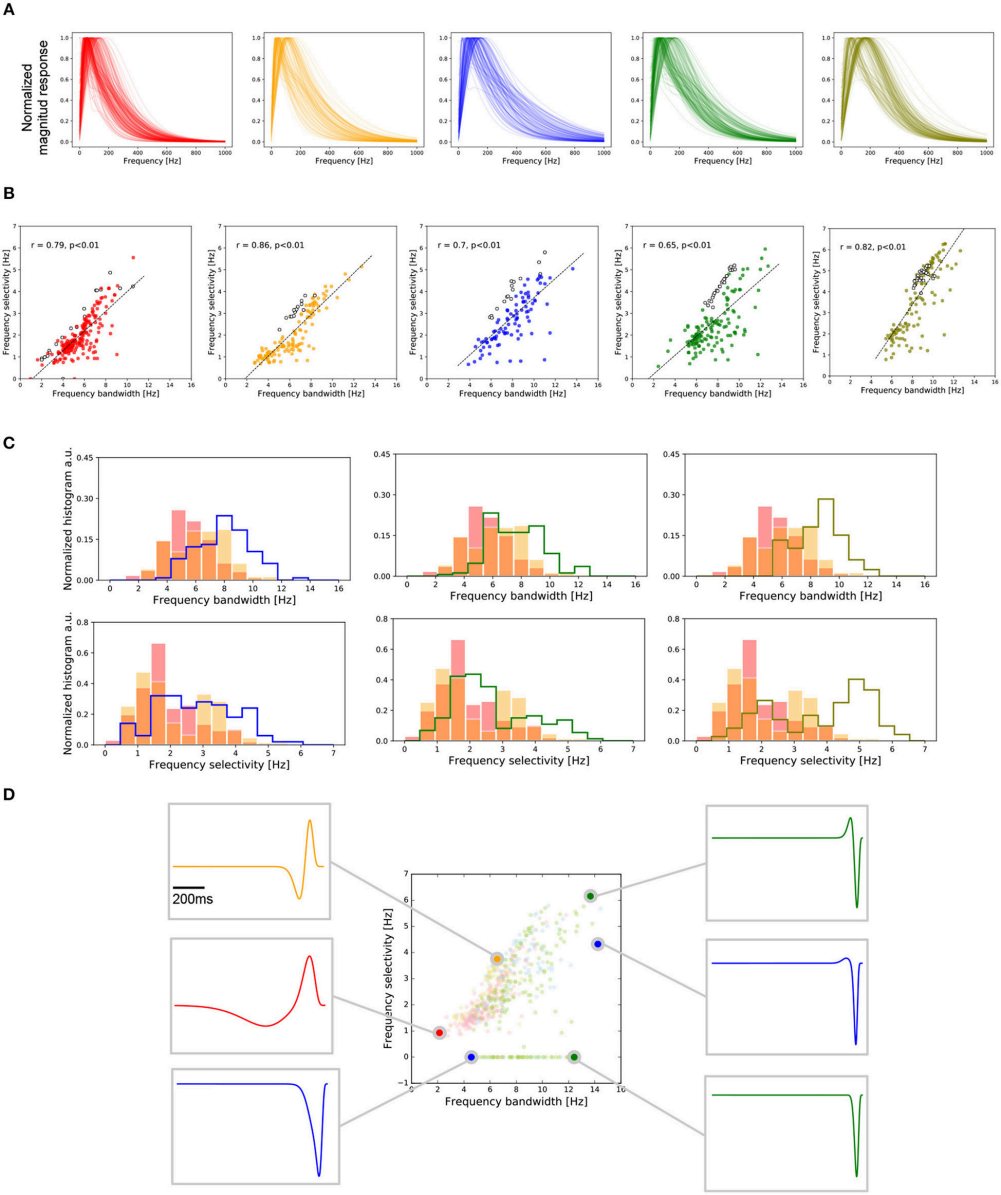
Frequency selectivity in central and peripheral RGCs. **(A)** Frequency response of the RGCs, temporal profiles of cells from the two central retina preparations (red and orange) and from the peripheral retina preparations (blue, green, and olive). **(B)** Covariation of the temporal frequency bandwidth and frequency selectivity for each retina preparation for all the RGCs recorded. Each preparation is accompanied by the respective regression indicating the level of correlation between these two values. **(C)** Histograms of the frequency bandwidth (top) and frequency selectivity (bottom) of all the recorded RGCs in the five retina pieces. Each column compares the centers against each of the peripheries. **(D)** Condensed representation of the five preparations analyzed. For the six selected, we show the temporal profile matching the frequency bandwidth and frequency selectivity values. Frequency selectivity is related to the cell biphasic-index. On the other hand, the response latency given by peak-time and zero-cross parameters is related to frequency bandwidth.

Both temporal frequency selectivity and frequency bandwidth were extracted from the temporal frequency response of each RGC obtained using the Fourier transform of the RF transient responses expression described in (1). Figure 5A shows the power spectrum density of the Fourier transform for each RGC, normalized to the same peak amplitude, for recordings from central and peripheral regions.

For each retina preparation, the relation between the temporal frequency selectivity and frequency bandwidth is represented in Figure 5B. OFF cells are represented by filled colored circles, while ON cells appear in empty circles. We observed a high correlation between these two parameters, as illustrated through the regression fit for each set of data.

Central RGCs have responses with smaller temporal frequency bandwidth compared to peripheral RGCs. Figure 5C (top) shows the histograms of the temporal frequency bandwidth for the two central preparations (red and orange data) contrasted with the same parameter observed on each preparation from the peripheral retina. We found no statistical difference between the center or the peripheries (KS, *p* > 0.001). Nevertheless, peripheries differed significantly from both centers (KS, *p* < 1e-7). Similarly, central RGCs had responses with smaller temporal frequency selectivity compared to peripheral RGCs (KS, *p* < 1e-5), as shown in Figure 5C (bottom).

We also wanted to match temporal frequency properties with the parameters in the temporal domain (see Figure 5D). For this we manually selected six cells located at the boundaries of the frequency region covered by the *Octodon degu*'s retina, obtained from the five experiments analyzed. This analysis allowed us to conclude that large zero-cross values in the temporal domain are associated with small frequency bandwidths. Similarly, small zero-cross values are related to large frequency bandwidths. Regarding frequency selectivity, we can observe that it is correlated with the biphasic index of the temporal response. Cells with a high biphasic index have a higher frequency selectivity compared to RGCs with a low biphasic index.

### 3.4. Isolating a Fast-Biphasic OFF Subtype

**Figure 6:**
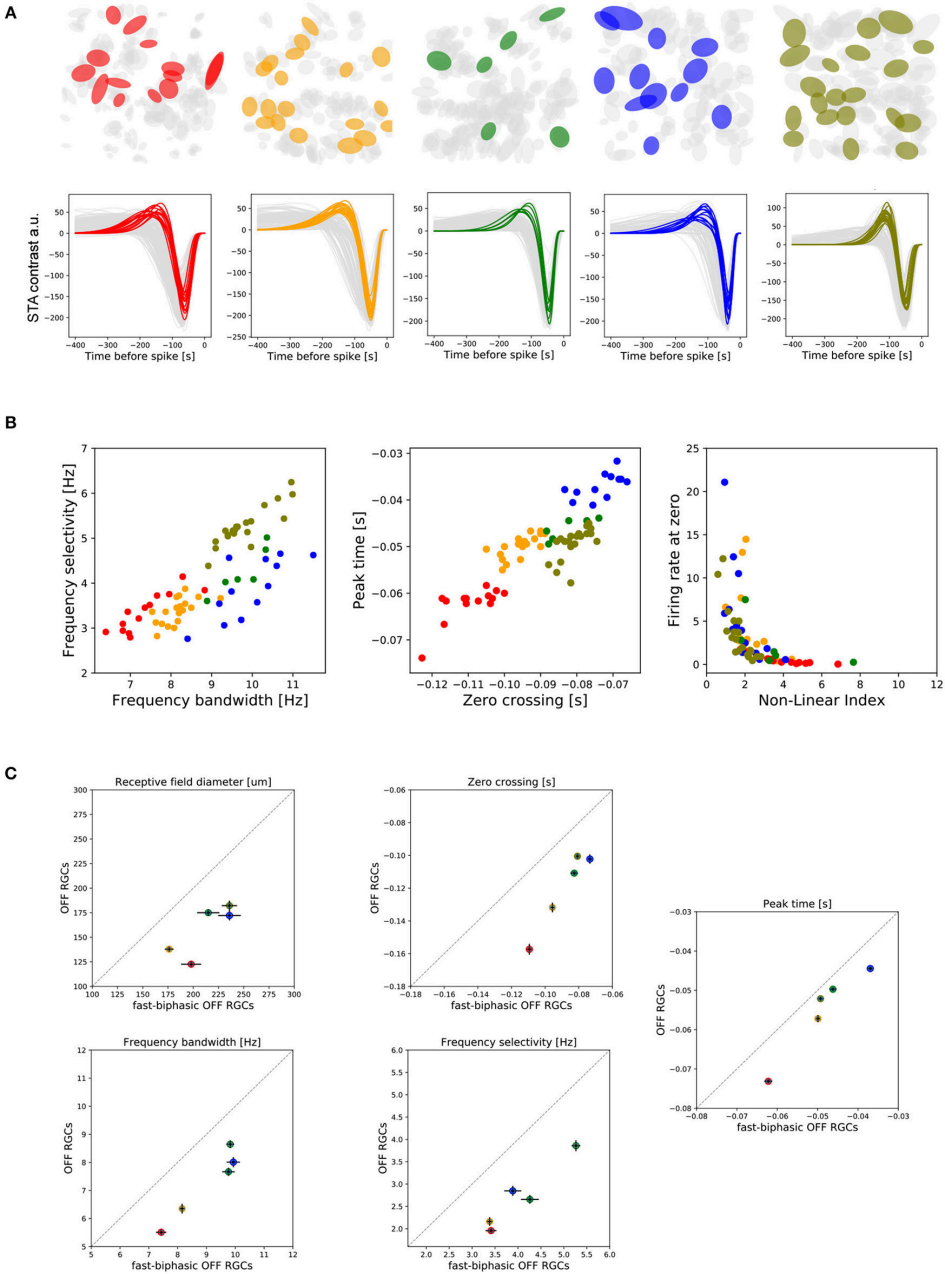
Functional properties of fast-biphasic OFF-RGCs. **(A)** Selected fast-biphasic OFF-RGCs from each retina piece studied (two from central and three from peripheral retina regions). **(B)** Left: Covariation of temporal frequency bandwidth and temporal frequency selectivity of fast-biphasic OFF-RGCs. Middle: Covariation of zero-crossing and peak frame parameters for fast-biphasic OFF-RGCs. Right: Covariation of non-linear index and firing rate measured when the linear estimated response is zero. **(C)** Two-D charts comparing functional properties of fast-biphasic OFF-RGCs with the entire population of OFF cells recorded. This comparison was done for RF sizes, zero-crossing, peak-frame, temporal frequency bandwidth, and temporal frequency selectivity parameters. Filled circles represent the population mean, while the error bars correspond to the Standard Error of the Mean (SEM).

We wanted to focus our analysis on a single type of RGC, the fast-biphasic OFF-RGC subtype. To do this, and following a similar selection criteria as Chichilnisky and Kalmar (2002), Deny et al. (2017), and Manookin et al. (2018), we isolated a single population considering: (i) a RF diameter in the 70th percentile, (ii) high biphasic-index, and (iii) a short zero-crossing parameter. The RGCs selected using these criteria are shown in Figure 6A.

In the central retina, fast-biphasic OFF-RGCs exhibit lower temporal frequency selectivity (KS, *p* < 0.01) and lower temporal frequency bandwidth (KS, *p* < 0.0001) than do their peripheral counterparts. In Figure 6B (left) we show the covariation of the two frequency characterization parameters. Similarly, fast-biphasic OFF-RGCs located in the central retina have larger zero-crossing values (KS, *p* < 1e-5) and larger peak-time values compared to peripheral cells of the same subtype, with the exception of one center and periphery which presented no significant differences (KS, *p* > 0.01—see Figure 6B middle). Looking at the non-linear index, with only a few exceptions, we observed no significant differences between the central and peripheral neurons of the same subtype.

In Figure 6C we compared the functional characteristics of the fast-biphasic OFF-RGC population compared to the entire OFF-RGC recorded. Filled colored circles represent the population mean, while vertical/horizontal error bars represent the Standard Error of the Mean (SEM). A comparison between all the spatio-temporal parameters studied here can be found in the Supplementary Figure 1.

### 3.5. Responses to Light Flashes

In addition to the analysis done using checkerboards, we also considered light-flash responses for the same set of cells. The light-flash stimulus was set at the beginning and it consisted of 30 repetitions of 400 ms ON and 1,000 ms OFF pulses. We only considered RGCs with a valid receptive field obtained with STA.

**Figure 7:**
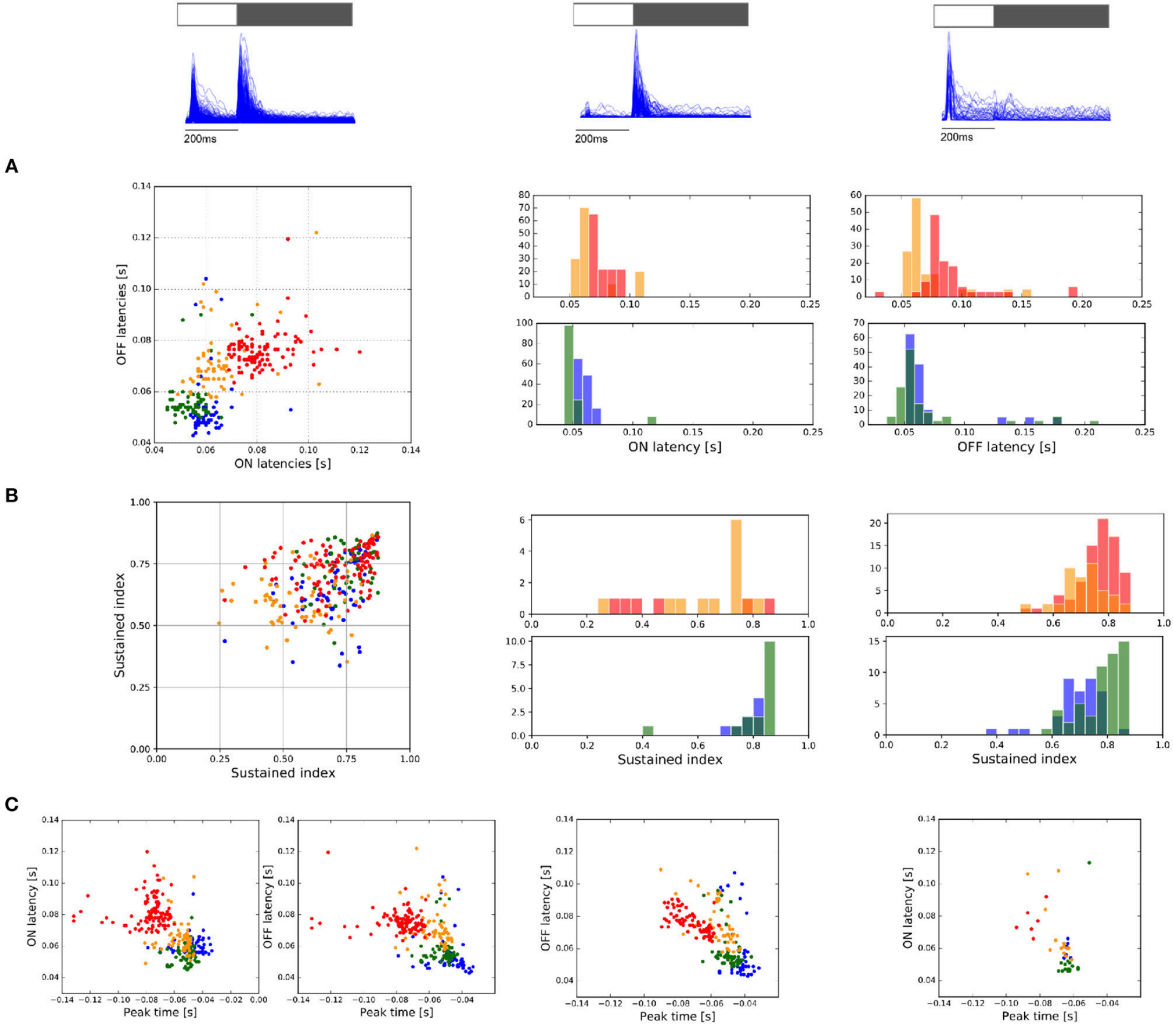
Response to ON and OFF light flashes. **(A)** ON and OFF latency responses measured for ON and OFF light flashes, respectively. From left to right, response of ON-OFF, OFF, and ON cells. **(B)** Sustained index (SI) as a manner to indicate if the response to light-flashes is more transient (SI = 1) or sustained (SI = 0). From left to right, values encountered for ON-OFF, OFF, and ON cell populations. **(C)** Comparison of the response latency encountered for light flashes with the peak-time parameter obtained with STA estimation. From left to right, values encountered for ON-OFF, OFF, and ON cell populations.

RGCs were separated into three cell populations according to their response to light-flashes, ON-OFF, OFF, or ON (see Figure 7). The response of each cell to the 30 repetitions was collapsed in a single PSTH signal, from which we obtained the response latency (time between the stimulus onset to the maximal cell response) and the sustained index (SI), which represents the time course response, either sustained (SI = 0) or transient (SI = 1). Figure 7A shows the response latency encountered in all the experiments analyzed, observing in most of the case central cells with the largest response latency value and a correlation between ON and OFF response latency for ON-OFF cells (first column). The only response where we did not observe differences with the periphery was the response latency of OFF cells. Similarly, the analysis of the SI did not show significant differences between the center and periphery for ON and OFF cell populations (Figure 7B). Nevertheless, statistical differences are observed between the SI values of ON-OFF responses between center and periphery (KS, *p* < 0.05).

As we considered cells with valid RFs, we correlated the response parameters obtained with light-flashes vs. temporal parameters obtained with STA analysis (see Figure 7C). Interestingly, isolated retina preparations only exhibited correlations between these two values for OFF cells (*r*>−0.6). Moreover, if we consider all the preparations as a whole, we observe correlations between these two parameters for all the responses (Figure 7C from left to right, *r* = −0.57, *r* = −0.53 and *r* = −0.63—ON cells were not considered).

### 3.6. Simulated Retina Response in a Real Scenario

**Figure 8:**
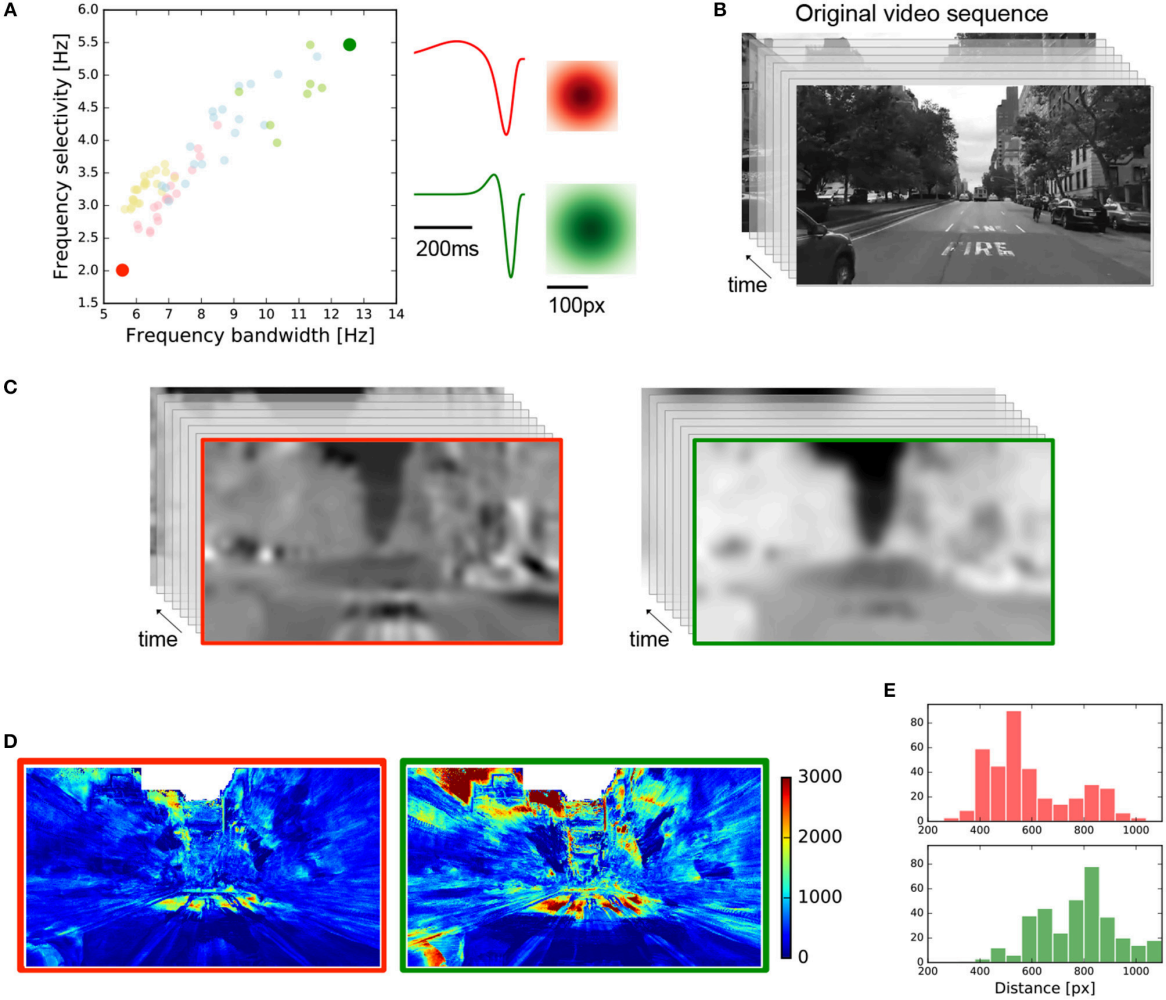
Simulated linear retina response in a real scenario for two fast-biphasic OFF-RGCs. **(A)** Two fast-biphasic OFF-RGCs were selected from the central and peripheral retina regions, at opposite sides of the frequency response chart. Spatial and temporal profiles are shown on the right. **(B)** Input video sequence from New York City used in this simulation (640 x 480 pixels, 200 frames, 60 fps). **(C)** Resulting videos obtained after the spatio-temporal convolution of the selected RGCs RF with the input video. Red/green for central/periphery RGCs, respectively. **(D)** Images representing the mutual information between the input video and the resulting videos after convolution. **(E)** Histograms quantify the mean mutual information value contained at a given distance from the center of the image. The red histogram (top) concentrates the information at lower distance values, while the green histogram (bottom) does so at larger distances.

Using the spatial and temporal profiles observed in the central and peripheral retina of the *Octodon degu* for the fast-biphasic OFF-RGCs, we asked whether their functional properties have a computational and therefore ecological meaning. To do so, we selected two cells at the opposite side of temporal frequency bandwidth and frequency selectivity chart, as shown in Figure 8A, represented as red (center) and green (ventral periphery) filled circles. These two selected points have the temporal profile and RF sizes shown on the right.

A video sequence of an approaching scene in New York City (Figure 8B 640 x 480 pixels, 200 frames, 60 fps) was used as input, and was convolved by the two selected cells. The input video was linearly filtered by each of the RGC receptive fields shown in Figure 8A, obtaining the responses shown in Figure 8C. For each pixel of the resulting videos, we extracted its temporal variation as a vector, and we computed the mutual information (see section 2) between this vector and the one obtained, for the same position, from the original video, having a value for each spatial location of the video. This procedure allowed us to detect the spatial regions of the video that were mainly encoded by the fast-biphasic OFF-RGCs selected. Figure 8D shows the results obtained for each case, where it is possible to observe that the central retina (left) mainly encodes the information located at the center of the image (which is mostly static), while the peripheral retina (right) cares about the moving parts which are primarily located at the lateral borders of the original video sequence. To quantify this, we computed the mean mutual information obtained for a given distance from the center of the image and obtained the red (center) and green (periphery) histograms shown in Figure 8E. These histograms show that for the central RGCs most of the information is located at lower distances from the image center, while for the peripheral RGC the information concentrates at higher distances.

## 4. Discussion

A comparative survey of the density distribution of RGCs as a function of retina eccentricity would put our results in context. The topographic distribution of RGCs shows, e.g., a high concentration for the central area for humans (fovea) (Curcio and Allen, 1990), mice (Dräger and Olsen, 1981; Salinas-Navarro et al., 2009a), and rats (Salinas-Navarro et al., 2009b). A study in marmoset reported 17 different RGC types, where interestingly the central retina is dominated by two of these types (parasol and midget), a characteristic that is not replicated in the periphery (Masri et al., 2016). The variety of morphological distributions of RGCs supports the existence of the even larger number of functional channels reported (Demb and Singer, 2015; Sanes and Masland, 2015; Baden et al., 2016).

Here we provide evidence supporting differential functional characteristics of RGCs as a function of retinal eccentricity in a diurnal rodent (*Octodon degus*). Similarly to other species, studies in this animal model have reported an increased cell density of photoreceptors (M and UV cones) and RGCs in a horizontal band around the optic nerve (Jacobs et al., 2003; Vega-Zuniga et al., 2013), suggesting a possible retinal specialization for central vs. peripheral retinal regions.

To investigate this, we analyzed the functional characteristics of central and peripheral retina patches. Using the functional characterization of RGC linear RFs and light-flash responses, we compared spatial and temporal characteristics and the asymmetries observed between different RGC populations. Additionally, we isolated a population of fast-biphasic OFF-RGCs that were selected considering the RF diameter and temporal response dynamics. This RGC subtype also evidenced functional differentiation at different eccentricities.

Diurnal rodents, exhibit interesting properties, such as a high number of cone photoreceptors, 30–40% (Bobu et al., 2008; Gaillard et al., 2008; Sa¨ıdi et al., 2011), mainly dominated by L-cones (90%) arranged in a horizontal steak (Rocha et al., 2009), and a more developed visual cortex (Campi and Krubitzer, 2010). In this study we report low percentage of ON cells, encountered either by checkerboard or light-flash stimuli (10–15%), which seems to be lower than the standard OFF/ON balance of 1.7 reported at the level of bipolar and RGCs in rabbits, rats, monkeys, or humans (Morigiwa et al., 1989; Dacey and Petersen, 1992; Devries, 2000; Chichilnisky and Kalmar, 2002). This is especially different compared to the balance of 1 encountered in mice under the same technique. This low presence of ON cells in the *Octodon degu* has been previously reported (Palacios-Muñoz et al., 2014), and it seems to be a property of diurnal rodents. It is still an open question if this light-response balance observed at the RGC level has consequences on the functional computations performed by the retina. Considering diurnal rodents have a large number of cones, together with a more developed visual cortex compared to nocturnal rodent it makes this animal model suitable for investigating retina and cone pathophysiology.

### Computation of Functional Types

Cell characterization relies on several of the cell's features including morphology, physiological responses, and molecular properties, which remains a challenging problem (Armañanzas and Ascoli, 2015). The authors in Sanes and Masland (2015) establish a series of criteria to identify a RGC type: uniform morphology, comparable gene expression, regular tiling, and uniform physiological properties. In our study, similar to the cell type identification performed by Zeck and Masland (2007), Deny et al. (2017), and Manookin et al. (2018), we use the uniform physiological property criterion to isolate a fast-biphasic OFF-RGC type based on the response obtained from checkerboard stimulus. Further analysis could also be done to refine or to extend the functional types encountered, for instance, considering motion and speed processing capabilities (see, e.g., Ben-Simon et al., 2012). The animal model studied here has a low percentage of ON-RGCs, especially in central regions and, importantly, this finding is only present using checkerboard stimulus. The small number of ON-RGCs recorded did not allow us to apply the same selection criteria for the ON population. Interestingly, a significant part of the ON-OFF cells reported in the light-flash stimulus characterization were classified as OFF cells when a white-noise stimulus was presented. This effect could be associated with the fact that ON-OFF cells in this animal model have a faster and stronger OFF response than ON. Additionally, this could also be associated to scotopic or photopic regimes, where it has been shown that light intensity changes the cell property to generate ON or OFF light responses (Tikidji-Hamburyan et al., 2014). Besides, even if this effect is observed, we analyzed the white-noise response to estimate linear spatio-temporal receptive fields as a standard and validated procedure used to characterize functional properties of cells in several animal models (Chichilnisky, 2001; Chichilnisky and Kalmar, 2002; Segev et al., 2005; Baden et al., 2016).

### Functional Differences Between Center and Periphery

Global analysis revealed an essential functional differentiation between central and peripheral RGCs. Moreover, these differences were also emphasized in the population subtype studied, the fast-biphasic OFF-RGCs. In the periphery, RGCs had larger RF sizes compared to central RGCs, suggesting large integration field areas (Figures 2, 6). Similar results were reported in the archer fish, where RFs at the central stream has a resolution comparable to photoreceptors (Ben-Simon et al., 2012). The increase of the RF diameter for large eccentricities could also be associated with the size of the RGC dendritic field. For instance, in non-human primates, there is a positive correlation between eccentricity and the dendritic field diameter (Chichilnisky and Kalmar, 2002).

Regarding the response dynamics, peripheral RGCs had faster (peak-time) and shorter (zero-crossing) time course responses compared with the central ones (Figure 3). We did not observe differences at the level of the biphasic index. In primate retina, Sinha et al. (2017) isolated midget ganglion cells observing that cell dynamics change along eccentricity, with cells in the periphery showing faster responses than those at the center. The difference in the response kinetics between different retina regions could be due to different inhibitory mechanisms, which have been demonstrated to play a role in shaping kinetics (Isaacson and Scanziani, 2011; Jadzinsky and Baccus, 2013). By using frequency analysis, we observed that fast and short temporal responses were related to a broader frequency bandwidth and a higher frequency selectivity (Figure 5). In particular, the fast-biphasic OFF-RGCs studied here (see Figure 6B), bounded regions of selectivity for central and peripheral cells. These findings are entirely consistent with the characteristics of higher areas of the visual system. For instance, Orban et al. (1986) compared central V1 and V2 visual areas vs. peripheral V1 regions in macaque monkeys, reporting that RF size and speed selectivity of V1 neurons also increase with eccentricity, which is mostly determined by the temporal frequency selectivity.

Considering the entire ON and OFF populations found using checkerboard stimulus, we observed no significant differences between them concerning linear/non-linear responses. Nevertheless, it has been reported in the literature that the linearity of their responses differs. For instance, Chichilnisky and Kalmar (2002) showed ON cells with nearly linear light responses whereas OFF cells acted more like rectifiers. Similarly, looking for the underlying mechanism to justify this behavior, Liang and Freed (2010) reported that input currents of OFF-RGCs were more rectified than those of ON-RGCs. This asymmetry may be an adaptation to natural scenes, which have more contrast levels below the mean than above. The differences between our findings and those reported in the literature could be mainly attributed to the type of ON cell observed, and the variations associated with the animal model used in each study.

### Functional Asymmetries Between ON and OFF RGC Populations

We compared the functional asymmetries between ON and OFF cell populations within each retina preparation and considered all the RGCs recorded. Regarding spatial characterization, the central pieces of the retina did not show differences between RF sizes for ON-RGC, and OFF-RGCs, and only one of the preparations from the periphery showed that RF diameters for the ON population were larger than those for the OFF population (Kolmogorov-Smirnoff, *p* < 0.02). Regarding temporal characterization, the temporal response of ON-RGCs, compared to OFF-RGCs varied along different retinal regions, and we systematically observed that OFF cells were faster (peak-time parameter) than ON cells. Nevertheless, observing the zero-crossing parameter related to the response duration, we noted a tendency of ON-RGCs to have shorter temporal courses compared to OFF-RGCs in almost all the samples analyzed (see Figure 4). Concerning the biphasic index, we observed a strong bias between ON-RGCs and OFF-RGCs populations, with ON cells having higher biphasic-index values compared to OFF-RGCs. Chichilnisky and Kalmar (2002) previously reported functional differences between ON-RGCs, and OFF-RGCs, kinetics, finding that in non-human primates ON cells had faster response kinetics than OFF-RGCs and that ON-RGCs had RF sizes 20% larger than OFF-RGCs.

Similarly, Zaghloul et al. (2003) investigated in the guinea pig the origins of this asymmetry, finding significant differences in the synaptic inputs between ON-RGC and OFF-RGC populations.

Using a light-flash stimulus, we observed a variety of responses where ON-OFF cells from the periphery exhibit both ON responses faster than OFF responses, and vice versa. For the central preparations we observed the same behavior. Similar to the results obtained with the checkerboard, we attribute this result to the different cell types gathered by each retina preparation.

The asymmetries observed between the temporal responses of ON and OFF populations can be studied in the temporal frequency domain. In general, we found that the ON population had higher frequency selectivity and higher bandwidth compared to OFF-RGCs (see Figure 5). We observed several RGCs acting as temporal low-pass filters, but this property was only present in OFF cells, not observed in ON cells. Considering that speed can be computed as temporal and spatial frequency selectivities, we can conclude that greater RF sizes and higher temporal frequency selectivities (ON properties) exhibit a selectivity to higher speeds compared to cells with lower temporal frequency selectivities (OFF-RGCs).

Nevertheless, it seems that the ON population presents a selective deficit in carrying information about moving stimuli, a deficiency that is not observed in the OFF-RGCs population (see Nichols et al., 2013). This finding is opposite to that reported by Leonhardt et al. (2016), in Drosophila, where ON cells respond maximally to slower velocities compared to OFF cells, suggesting that ON and OFF populations encode speed information in a combined manner: at slow velocities, the coding is driven by ON cells, while at high velocities it is driven by OFF cells. These results could probably be combined with our findings concerning the temporal or spatial frequency bandwidth, where ON and OFF RGC populations present different distributions.

### Ecological Justification of Segregated Regions and the Advantages of This Diversity

Different functionalities of the central and peripheral vision are attributed to ecological needs involved in behavior and survival. Central vision encodes fine details of the visual scene, while peripheral vision is related to coarse spatial and temporal frequency information mostly involved in generating alert messages needed for survival. Furthermore, a recent study in mouse retina showed that the encoding differences along retinal regions are not only between center and periphery, but also between dorsal and ventral areas (Warwick et al., 2018). Behavioral studies in humans related to object recognition revealed a better performance when the object is placed within 1-2° of the visual field (Musel et al., 2013), similar to the case for facial recognition (Arcaro et al., 2009). Nevertheless, humans confronted with natural scenes in their peripheral visual regions show greater fMRI responses, suggesting combined processing strategies for understanding the visual world (Nasr et al., 2011; Loschky et al., 2017). Interestingly, using a deep neural network the authors in Wang and Cottrell (2017) have shown that information coming from peripheral vision is as crucial for scene recognition as central vision.

A diversity of visual strategies can be created through the articulation of a variety of functional RGCs types located in the retina. Predictive coding theory hypothesizes that the role of neural encoding is to remove predictable information from the environment and to leave only the information that cannot be predicted. The work of Nirenberg et al. (2010) goes in this direction, suggesting that heterogeneity in RGC types is needed for predictive coding in different kinds of environments. Moreover, Gjorgjieva et al. (2014) recently reported that optimal coding in the retina is reached not by isolated ON or OFF neuronal populations, but by a mixture of ON/OFF neurons, suggesting a combined coding strategy.

## Author Contributions

MJ-E designed the experiments, analyzed data and wrote the paper. CR, MO, and RH analyzed data. JA and CI performed the retinal experiments. AP edited the paper.

### Conflict of Interest Statement

The authors declare that the research was conducted in the absence of any commercial or financial relationships that could be construed as a potential conflict of interest.

## Supplementary Material

Supplementary Figure 1 Comparison between all the spatiotemporal parameters studied in this article. The relation between two parameters is represented as scatter-plots for the upper triangular matrix, and as level curves for the lower triangular matrix. The color code used is the same for all the article: the two centers are shown in red and orange; while the two peripheries are represented in green and blue.
