# Supplementary figures and images for: Characterization of Retinal Functionality at Different Eccentricities in a Diurnal Rodent
Source: Front Cell Neurosci. 2018 Dec 3;12:444. doi: 10.3389/fncel.2018.00444 (PMC6287453; doi:10.3389/fncel.2018.00444)

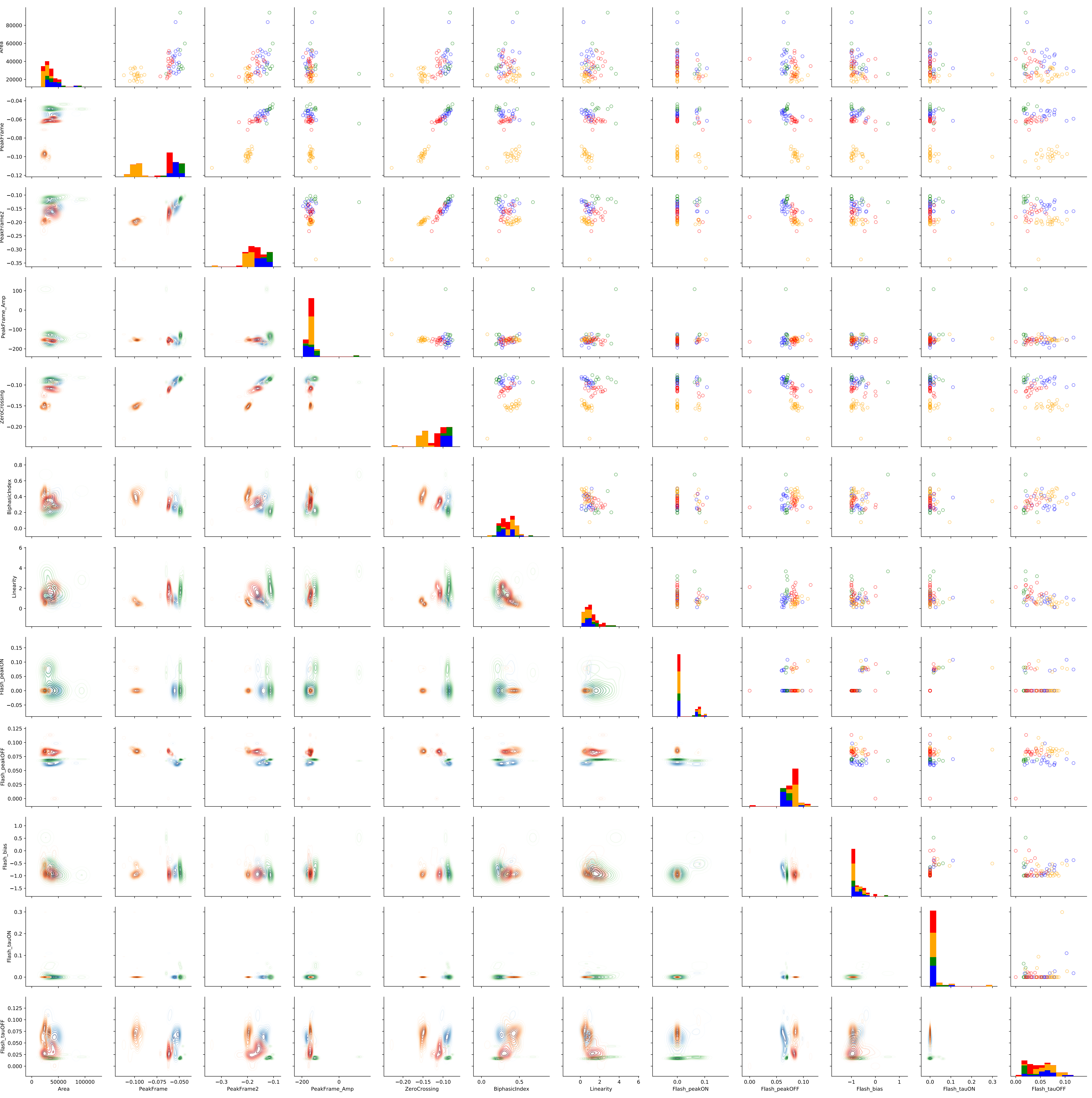

Supplement: Supplementary Figure 1 — Comparison between all the spatiotemporal parameters studied in this article. The relation between two parameters is represented as scatter-plots for the upper triangular matrix, and as level curves for the lower triangular matrix. The color code used is the same for all the article: the two centers are shown in red and orange; while the two peripheries are represented in green and blue. [file Image_1.pdf]
